# Combined cell surface carbonic anhydrase 9 and CD147 antigens enable high-efficiency capture of circulating tumor cells in clear cell renal cell carcinoma patients

**DOI:** 10.18632/oncotarget.10979

**Published:** 2016-08-01

**Authors:** Shijie Liu, Zuhong Tian, Lei Zhang, Shuang Hou, Sijun Hu, Junshen Wu, Yuming Jing, Huimin Sun, Fei Yu, Libo Zhao, Ruoxiang Wang, Hsian-Rong Tseng, Haiyen E. Zhau, Leland W.K. Chung, Kaichun Wu, Hao Wang, Jason Boyang Wu, Yongzhan Nie, Chen Shao

**Affiliations:** ^1^ Department of Urology, Xijing Hospital, The Fourth Military Medical University, Xi'an, Shaanxi 710032, China; ^2^ State Key Laboratory of Cancer Biology & Xijing Hospital of Digestive Diseases, The Fourth Military Medical University, Xi'an, Shaanxi 710032, China; ^3^ Department of Epidemiology, The Fourth Military Medical University, Xi'an, Shaanxi 710032, China; ^4^ Department of Molecular and Medical Pharmacology, Crump Institute for Molecular Imaging, California Nanosystems Institute, University of California, Los Angeles, CA 90095, USA; ^5^ Beijing National Laboratory for Molecular Sciences, Key Laboratory of Molecular Nanostructure and Nanotechnology, Institute of Chemistry, Chinese Academy of Sciences, Beijing 100190, China; ^6^ Uro-Oncology Research Program, Department of Medicine, Samuel Oschin Comprehensive Cancer Institute, Cedars-Sinai Medical Center, Los Angeles, CA 90048, USA; ^7^ CAS Key Laboratory for Biological Effects of Nanomaterials and Nanosafety, National Center for Nanoscience and Nanotechnology, Beijing 100190, China; ^8^ Department of Pharmaceutical Sciences, College of Pharmacy, Washington State University, Spokane, WA 99202, USA

**Keywords:** carbonic anhydrase 9, CD147, circulating tumor cells, renal cell carcinoma

## Abstract

Circulating tumor cells (CTCs) have emerged as promising tools for noninvasive cancer detection and prognosis. Most conventional approaches for capturing CTCs use an EpCAM-based enrichment strategy, which does not work well in cancers that show low or no expression of EpCAM, such as renal cell carcinoma (RCC). In this study, we developed a new set of cell surface markers including CA9 and CD147 as alternative CTC-capture antigens specifically designed for RCC patients. We showed that the expression of both CA9 and CD147 was prevalent in a RCC patient cohort (n=70) by immunohistochemical analysis, with both molecules in combination covering 97.1% of cases. The NanoVelcro platform combined with CA9-/CD147-capture antibodies demonstrated significantly higher efficiency for capturing both CTC-mimicking renal cancer cells and RCC CTCs in peripheral blood, compared to the conventional EpCAM-based method. Using immunofluorescence cytological validation at the single-cell level, we were able to identify bona fide CTCs in RCC patient blood following the well-accepted criteria in our CTC-capture system. We further demonstrated a significant association of CTC numbers as well as the CTC expression status of Vimentin, a mesenchymal marker, with disease progression, including pathologic features and clinical staging. These results provide new insights into developing novel, effective targets/approaches for capturing CTCs, making CTCs a valuable tool for improved cancer detection, prognosis and treatment in RCC.

## INTRODUCTION

Circulating tumor cells (CTCs) are cancer cells that invade blood circulation, eventually leading to systemic dissemination at distant sites [1]. Understanding the biological nature of CTCs is the key to the diagnosis, prognosis and treatment of cancer metastasis. Recently, the clinical relevance of CTCs in metastatic cancers has been clearly demonstrated in multiple types of advanced cancer including breast cancer, prostate cancer and colon cancer, where CTC detection has been significantly enhanced by the development of EpCAM-based enrichment strategies [2-4].

The epithelial cell adhesion molecule (EpCAM), a cell surface glycoprotein highly expressed in epithelial cancer cells, currently serves as the major capture antigen for CTC detection [5, 6]. However, EpCAM is only partially expressed in certain types of cancers, leading to inefficient capture and the escape of CTCs from EpCAM-based detection, which is further exacerbated by the fact of tumor heterogeneity [7, 8]. In addition, the use of EpCAM-based enrichment techniques likely leads to the failure to detect CTC populations that have undergone epithelial-mesenchymal transition (EMT), with the loss of more epithelium-like CTCs [9, 10]. The limitations of EpCAM-based capture methods indicate the urgent need to develop alternative cancer type-specific capture antigens for improved capture of CTCs.

In recent decades, the incidence of renal cell carcinoma (RCC) has risen steadily; kidney cancer, with the vast majority being RCC, was the 7^th^ leading cancer type in men in the Unites States in 2015 [11]. Successful management of RCC patients, particularly those with recurrent and metastatic RCC, largely relies on early detection and prognosis of disease progression. The noninvasive detection of CTCs in peripheral blood of patients has become a useful clinical tool in other cancers, such as breast cancer [12], but the conventional CTC-capture marker EpCAM is only expressed in 30-40% of RCC and thus is not an ideal capture antigen for the detection of CTCs in RCC patients [13]. Although other biomarkers, including p53, p21, HIF-1α, caveolin-1 and survivin, have been reported as potential prognostic biomarkers for RCC patients [14, 15], these markers are not located on the cell membrane. Therefore, improving the efficiency of CTC capture in RCC patients by developing alternative cell surface biomarkers remains a challenge.

By immunohistochemical (IHC) analysis of clinical RCC specimens, we recently found two surface biomarkers, carbonic anhydrase 9 (CA9) and CD147, which show excellent features as candidate capture antigens for RCC CTCs. CA9, a transmembrane member of the carbonic anhydrase family, is not expressed in healthy and benign renal tissues, but it is present in 91.2% of clear cell renal cell carcinomas (CCRCC), which make up 75% of RCC, by IHC analysis [16, 17]. CD147, a highly glycosylated member of the immunoglobulin superfamily expressed on the surface of many malignant tumors, such as ovarian cancer and bladder cancer [18-20], is also positively expressed in up to 88.7% of patients with advanced RCC [21]. In addition, recent studies using multiple antigens to capture CTCs suggest the possibility of using CA9 and CD147 together for more effective and sensitive capture of CTCs compared to use of a single molecule for RCC diagnostics and prognostics [22-25]. In this study, we demonstrated the use of CA9 and CD147 in combination as effective antigens for capturing CTCs in RCC patients with higher efficacy and sensitivity than the conventional EpCAM-based detection, suggesting the translational potential of our method for future development of CTC detection technologies for clinical applications to benefit RCC patients.

## RESULTS

### Validation of CTC-capture antigens

In this study, we used the NanoVelcro System, a nanostructured platform, to capture CTCs from 2 ml peripheral blood of each patient in a cohort including 76 RCC patients, 10 benign renal patients and 15 healthy volunteers (Table 1). The isolated CTCs were subsequently subjected to pathologic identification and cellular characterization. Owing to the advantages of its topographic interaction effect, this CTC-capture platform has been successfully used for the enrichment of CTCs in different types of cancer [26-29]. Given that clear cell renal cell carcinoma (CCRCC) is the dominant form accounting for about 75% of all RCCs [16], we focused on this specific RCC subtype in the present study.

**Table 1:** Characterization of 86 patients (renal cell carcinoma, n=76; benign, n=10) and 15 healthy volunteers registered in the CTC enumeration study

Subject category	n
Volunteer	15
Benign	10
Renal cell carcinoma (RCC)	76
**Subject gender**	**Age, median (range)**
Total	56 (16-78)
Men	53 (69.7)
Women	23 (32.8)
**RCC subtype**	**n (%)**
Clear cell renal cell carcinoma	76 (100%)
**Stage**	**n (%)**
I	29 (38.2%)
II	19 (25.0%)
III	16 (21.1%)
IV	12 (15.8%)
**Tumor location**	**n (%)**
Left kidney	36 (47.4%)
Right kidney	40 (52.6%)

**Figure 1:**
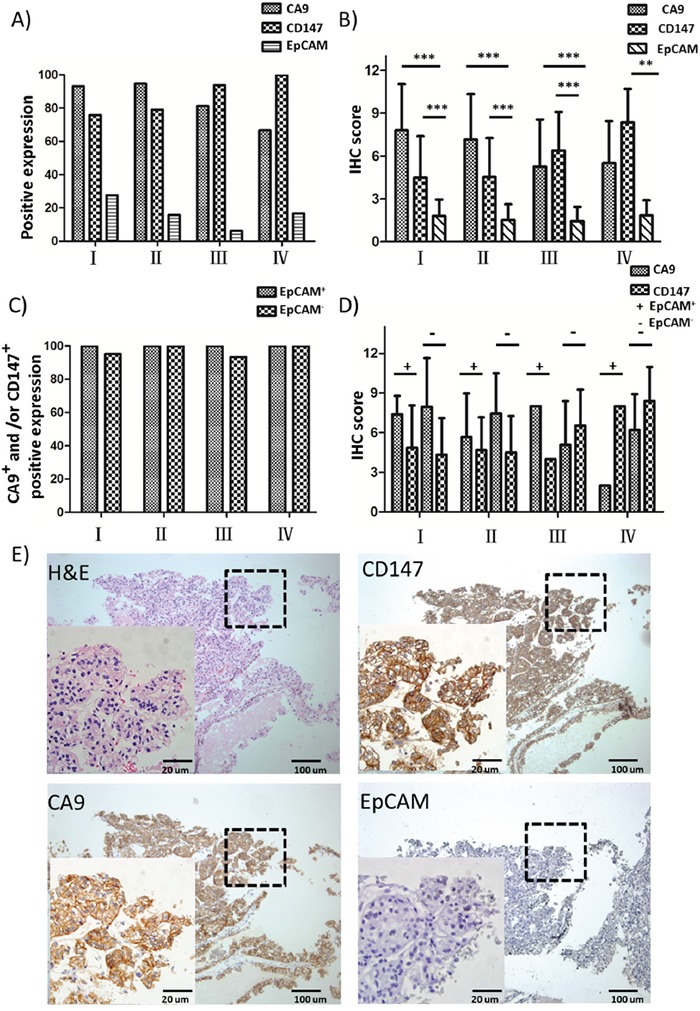
IHC analysis of EpCAM, CA9 and CD147 expression in RCC patient cohort **A.** Positive expression percentages of EpCAM, CA9 and CD147 in RCC patient samples of different clinical stages by IHC analysis (I, n=29; II, n=19; III, n=16; IV, n=6). **B.** IHC scores of EpCAM, CA9 and CD147 in RCC samples of (A). *** *p*<0.001. **C.** Positive expression percentages of CA9^+^ and/or CD147^+^ in EpCAM^+^ (I, n=8; II, n=3; III, n=1; IV, n=1) or EpCAM^-^ (I, n=21; II, n=16; III, n=15; IV, n=5) RCC patient samples of different clinical stages by IHC analysis. **D.** IHC scores of EpCAM, CA9 and CD147 in RCC samples of (C). **E.** H&E and IHC staining of EpCAM, CA9 and CD147 in representative EpCAM^-^ RCC patient tissues. The selected enlarged areas are indicated in dashed rectangles. Original magnification, 400× (enlarged) and 100× (original); scale bars: 20 μm (enlarged) and 100 μm (original).

We first evaluated the simultaneous expression of EpCAM, CA9 and CD147 in serial sections of tumor specimens collected from the RCC patient cohort spanning 4 different clinical stages by IHC analysis. EpCAM was only positively expressed in 18.6% of cases with a range of 6.3-27.6% in each clinical stage, and there was a declining trend of EpCAM positivity in patients with advanced-stage RCC (III and IV) compared to early-stage cases (I and II) (Figure 1A and Table 2). By contrast, CA9 and CD147 showed prevalent expression in 88.6% and 82.9% of cases, respectively (Table 2). Moreover, CA9 had a slightly higher rate of positive expression in early-stage (I and II) relative to advanced-stage (III and IV) cases, whereas CD147 was more positively expressed in stage III and IV cases than those with stages I and II (Figure 1A and Table 2). Therefore, CA9 and CD147 in combination complement each other and cover the entire cohort to a greater extent than either antigen alone. Indeed, 97.1% of cases showed positive expression of CA9, CD147 or both, with a range of 93.8-100% in each clinical stage (Figure 1A and Table 2). Notably, we showed positive expression of CA9 and/or CD147 in 96.4% of EpCAM^-^ cases as well as 100% of EpCAM^+^ cases (Figure 1C and Table 2). We also analyzed the parallel IHC expression of CA9, CD147 and EpCAM by a semi-quantitative method by taking into account both quantity of proportion of cells stained and staining intensity. Consistently, CA9 and CD147 demonstrated significantly higher IHC scores than EpCAM across all stages (Figure 1B), with the majority of IHC scores distributed in an EpCAM-independent manner in each stage (Figure 1D). In addition, as revealed by IHC staining, all three markers were expressed in tumor cell membrane with no expression in other types of cells, such as stromal cells, in the same areas (Figure 1E). These observations in aggregate suggest the potential of using CA9 and CD147 in combination as capture antigens to replace EpCAM for capturing CTCs with higher enrichment efficiency.

**Table 2:** Distribution of positive expression of EpCAM, CA9 and CD147 in the RCC patient cohort

	I	II	III	IV	Total
CA9^+^	27/29 93.1%	18/19 94.7%	13/16 81.3%	4/6 66.7%	62/70 88.6%
CD147^+^	22/29 75.9%	15/19 79.0%	15/16 93.8%	6/6 100%	58/70 82.9%
EpCAM^+^	8/29 27.6%	3/19 15.8%	1/16 6.3%	1/6 16.7%	13/70 18.6%
CA9^+^ and/or CD147^+^	28/29 96.5%	19/19 100%	15/16 93.8%	6/6 100%	68/70 97.1%
CA9^+^ and/or CD147^+^ in EpCAM^+^ group	8/8 100%	3/3 100%	1/1 100%	1/1 100%	13/13 100%
CA9^+^ and/or CD147^+^ in EpCAM^−^ group	20/21 95.2%	16/16 100%	14/15 93.3%	5/5 100%	55/57 96.4%

### Optimization of the NanoVelcro capture platform

**Figure 2:**
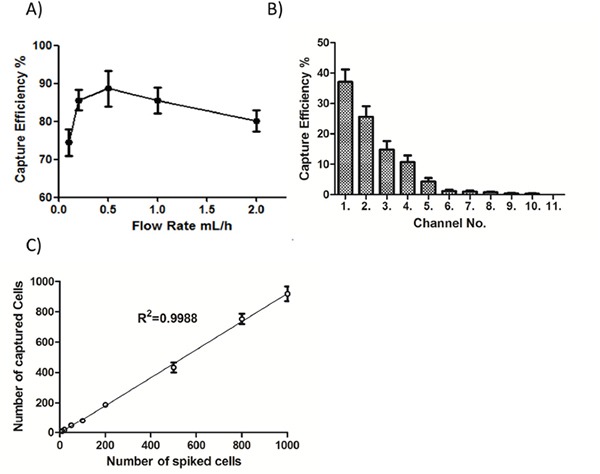
Optimization of the NanoVelcro system **A.** Cell-capture efficiency of the NanoVelcro Chip at flow rates of 0.1, 0.2, 0.5, 1, and 2 mL/h. Data represent the mean ± SD (n=3). **B.** The cell distribution and accumulative cell-capture efficiency in a NanoVelcro Chip assessed in PBS. **C.** Cell-capture efficiency with different spiked cell numbers ranging from 10 to 1000 cells/mL in PBS.

To optimize the working conditions of the NanoVelcro platform, we tested the capture efficiency at different flow rates (i.e. 0.1, 0.2, 0.5, 1 and 2 mL/h). As shown in Figure 2A, the highest capture efficiency was obtained at 0.5 mL/h, which was selected for subsequent capture of CTCs in patient blood. We also assessed the cell distribution on the channels of patterned silicon nanowire substrate, and the majority of captured cells were found in the first 10 channels among all 11 channels structured in the chip (Figure 2B). Finally, we spiked serial numbers of cultured renal cancer cells in saline to mimic the circulation of CTCs in blood, and demonstrated that the NanoVelcro system was capable of capturing these CTC-mimicking cells in a cell number-dependent linear manner (R^2^=0.9988, Figure 2C). Together, these studies optimized the CTC-capture conditions of the NanoVelcro platform in the present system, allowing a steady and reliable collection of CTCs in clinical scenarios.

### Combined CA9-/CD147-based capture of renal cancer cells and CTCs

**Figure 3:**
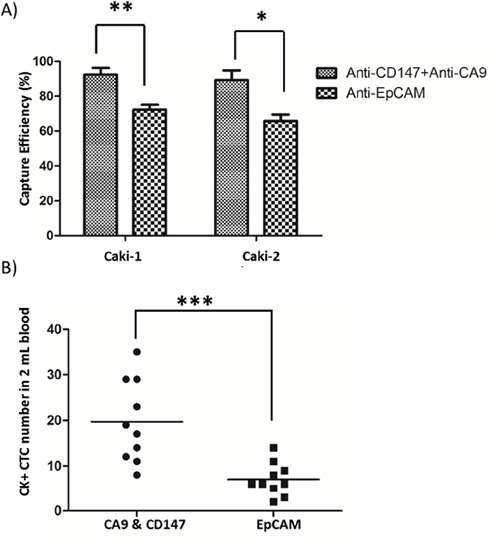
CTC capture by NanoVelcro Chips coupled with combined use of CA9 and CD147 as capture antigens **A., B.** Comparisons of capture efficiency between combined CA9-/CD147-based and EpCAM-based NanoVelcro Chips for CTC-mimicking Caki-1/Caki-2 renal cancer cells (A) or CTCs in peripheral blood of 10 RCC patients (B). * *p*<0.05, ** *p*<0.01, *** *p*<0.001.

**Figure 4:**
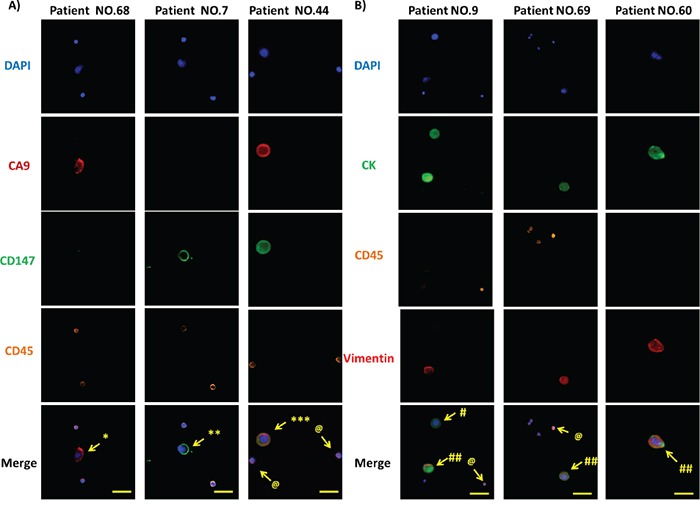
Immunofluorescence microscopic characterization of CTCs **A.** DAPI (blue), anti-CA9 (red), anti-CD147 (green) and anti-CD45 (orange) antibodies were used to stain nuclei, CA9, CD147 and WBCs, respectively. Images from 3 representative patient samples (No. 68, 7 and 44) are shown. * DAPI^+^/CA9^+^/CD45^-^ CTCs, ** DAPI^+^/CD147^+^/CD45^-^ CTCs, *** DAPI^+^/CA9^+^/CD147^+^/CD45^-^ CTCs, and @ DAPI^+^/CD45^+^ WBCs. Original magnification, 400×; scale bars: 50 μm. **B.** DAPI (blue), anti-pan CK (green), anti-CD45 (orange) and anti-Vimentin (red) antibodies were used to stain nuclei, epithelial cells, WBCs and mesenchymal composition of CTCs, respectively. Images from 3 representative patient samples (No. 9, 69 and 60) are shown. # DAPI^+^/CK^+^/CD45^-^/Vimentin^-^ CTCs, ## DAPI^+^/CK^+^/CD45^-^/Vimentin^+^ CTCs, and @ DAPI^+^/CD45^+^ WBCs. Original magnification, 400×; scale bars: 50 μm.

To examine the renal CTC-capture efficiency of using CA9 and CD147 as capture antigens, we first used the NanoVelcro platform to capture renal cancer cells from two representative human CCRCC lines, Caki-1 and Caki-2. Compared to the EpCAM-based enrichment approach, the combined use of CA9 and CD147 in parallel allowed the capture of both Caki-1 (*p*<0.01) and Caki-2 (*p*<0.05) cells with higher efficiency (Figure 3A). Next, we compared the efficiency of capturing clinical RCC CTCs between EpCAM- and CA9-/CD147-based enrichment strategies by the NanoVelcro system. We demonstrated a 181% increase of capture efficiency of cytologically validated CTCs in 2 mL blood when CA9 and CD147 were used in combination as capture antigens in comparison with EpCAM antigen (*p*<0.001, Figure 3B), which was consistent with the observations made by IHC in clinical RCC samples. We also confirmed a high percentage of CA9^+^ and/or CD147^+^ expression, with an average of 84%, on the cell membrane of CTCs (DAPI^+^/CD45^-^) captured by CA9-/CD147-based enrichment approach in 94% (31 out of 33) samples we examined, which was accompanied by positive staining of CA9 and CD147 in the corresponding RCC samples as revealed by IHC (Figure 4A and Table 3). The missing expression of CA9 and/or CD147 in 2 CTC samples (patients 22 and 27) was largely due to their low expression in the corresponding primary tumors where CTCs were considered to shed (Table 3). Although we compared the expression of CA9 and CD147 in isogenic pairs of primary tumors and CTCs from only a portion (33 out of 76) of our cohort due to limited quantities of patient blood we were able to collect for multiple analyses, the prevalent consistency of their expression patterns in these samples spanned 4 different clinical stages and thus, to a great extent, might represent the entire cohort. Collectively, these results support the idea of substituting EpCAM with combined CA9 and CD147 as capture antigens for improved capture efficiency and sensitivity in RCC.

**Table 3:** Numbers of CA9^+^ and/or CD147^+^ CTCs in 2 mL blood of select patients from the RCC patient cohort

Patient No.	Stage	Cell No. (DAPI^+^/CD45^−^)	Cell No. (DAPI^+^/CD45^−^/CA9^+^ and/or CD147^+^)	% (DAPI^+^/CD45^−^/CA9^+^ and/or CA147^+^)	IHC score (CA9)	IHC score (CD147)
1	I	11	9	82	12	3
2	I	16	14	88	9	2
4	I	20	18	90	6	9
6	I	9	9	100	9	0
8	I	25	20	80	8	12
9	I	15	14	93	9	1
14	I	28	23	82	9	6
15	I	6	5	83	8	9
16	I	11	9	82	12	1
19	I	9	8	89	6	3
20	I	25	23	92	12	3
22	I	2	0	0	3	1
25	I	16	13	81	9	4
27	I	5	0	0	0	4
29	I	13	10	77	9	8
31	II	11	9	82	3	3
34	II	21	19	90	9	6
35	II	16	13	81	12	3
39	II	17	15	88	9	3
41	II	11	9	82	1	8
43	II	28	19	68	4	4
44	II	21	15	71	3	12
45	II	17	14	82	9	8
49	III	23	19	83	9	4
51	III	16	13	81	12	3
55	III	20	19	95	8	4
59	III	25	18	72	2	9
60	III	17	12	71	4	6
61	III	21	17	81	3	9
62	III	19	16	84	9	8
63	III	34	33	97	6	6
67	IV	12	11	92	9	4
70	IV	8	7	88	8	9

### Cytological characterization of CTCs

To eliminate the potential interference of white blood cells (WBCs) with the captured CTCs, we enumerated and characterized CTCs at the single-cell level by immunofluorescence. We defined cells meeting all the following criteria under microscopy as bona fide CTCs: 1) cellular diameter between 13 μm and 50 μm, 2) positive cytokeratin (CK) expression for epithelial cell staining but negative CD45 expression for WBC staining, and 3) a >50% nuclear-cytoplasmic ratio for CTCs in contrast to that less than 30% for WBCs. We used DAPI, fluorescence-labeled anti-pan-CK and anti-CD45 antibodies to visualize nuclei, epithelial cells and WBCs, respectively. In addition to these cellular markers, we further introduced Vimentin, a mesenchymal marker, to characterize the mesenchymal state of captured CTCs, which is in line with emerging studies that demonstrated the ability of CTCs to exhibit dynamic changes in epithelial and mesenchymal composition as well as an association of mesenchymal CTCs with disease progression [30-33]. Following the CTC selection criteria cited above, we identified three different types of cells in the entire pool of captured cells from the patient cohort, including two types of CTCs, DAPI^+^/CK^+^/CD45^-^/Vimentin^+^ and DAPI^+^/CK^+^/CD45^-^/Vimentin^-^, and WBCs defined by DAPI^+^/CK^-^/CD45^+^. Of note, we were able to observe all three types of cells in a single patient sample, such as Patient 9, patient 60, patient 69 (Figure 4B). These results demonstrate the capability of the combined CA9-/CD147-based enrichment method to capture bona fide RCC CTCs from clinical blood samples.

### Clinical implications of CTCs in RCC

**Figure 5:**
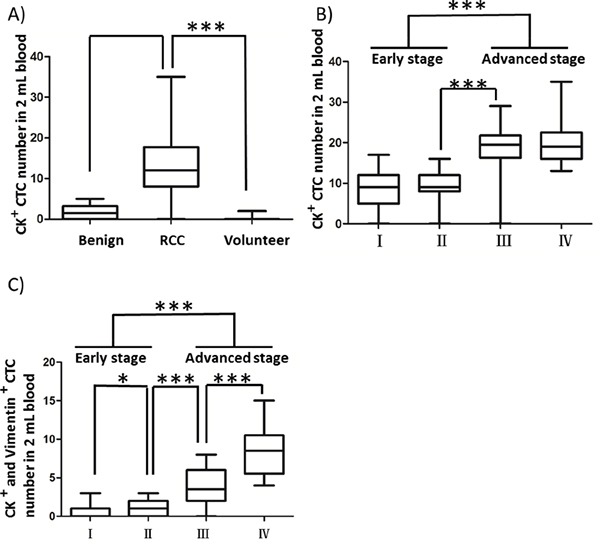
Correlation of CTC counts and CTC expression status of Vimentin with clinical indices in RCC patients **A.** Comparison of CK^+^ CTC counts in 2 mL peripheral blood from healthy volunteers, benign and RCC patients. Blood draw was conducted at the first visit for all registrants prior to surgery. **B, C.** Comparisons of numbers of CK^+^/CD45^-^ (B) and CK^+^/CD45^-^/Vimentin^+^ (C) CTCs captured in 2 mL blood from RCC patients across 4 different clinical stages. * *p*<0.05, ** *p*<0.01, *** *p*<0.001.

To examine whether the CTCs captured by the combined CA9-/CD147-based enrichment approach are clinically relevant, we sought to associate the numbers of cytologically validated CTCs with different clinical indices, including pathologic indication and clinical stage. We were able to detect CTCs in 94.7% of RCC patients (72/76). As shown in Figure 5A, the number of DAPI^+^/CK^+^/CD45^-^ CTCs captured in 2 mL RCC patient blood (12.8 ± 6.9, n=76) was significantly higher, with a 7.5-fold increase over that obtained from benign patients (1.7 ± 1.7, n=10), including angiomyolipoma, renal adenoma and fibroma. As expected, we did not detect DAPI^+^/CK^+^/CD45^-^ CTCs in 2 mL peripheral blood from 13 out of 15 healthy volunteers except 2 donors wherein one or two cells with potential malignancy were captured and characterized as DAPI^+^/CK^+^/CD45^-^ with cellular diameter falling in the range of 13-50 μm, which was technically due to the contamination of normal epithelial cells leaking into vacuum tubes during blood draw. Moreover, we showed that the number of DAPI^+^/CK^+^/CD45^-^ CTCs was associated with the clinical stages of RCC, with 1.2-fold more CTCs present in late-stage (III and IV) patient blood compared to those with early-stage (I and II) disease (Figure 5B and Table 4). By stratifying CTCs by Vimentin expression, we further demonstrated a significant correlation of DAPI^+^/CK^+^/CD45^-^/Vimentin^+^ CTC number with clinical stages (I-IV) in the RCC patient cohort (Figure 5C and Table 4), suggesting that the mesenchymal status of CTCs could serve as a potential prognostic factor for RCC patients. In conclusion, we showed that the numbers of RCC CTCs captured from peripheral blood by our CA9-/CD147-based enrichment methods were well associated with the disease progression of RCC patients.

**Table 4:** Numbers of CK^+^ and CK^+^/Vimentin^+^ CTCs in 2 mL patient blood of the RCC patient cohort

Numbers	I	II	III	IV
CK^+^	8.76 ± 4.11	9.32 ± 3.64	18.56 ± 5.96	20.42 ± 5.97
CK^+^/Vimentin^+^	0.55 ± 0.77	1.11 ± 1.02	3.81 ± 2.27	8.5 ± 3.15

## DISCUSSION

The detection of CTCs in genitourinary cancers, in particular prostate cancer and bladder cancer, has been widely investigated [4, 34]. In contrast, CTC research in RCC has shown little progress, which is due in large part to the lack of appropriate surface markers that can be used as capture antigens [35]. Currently, EpCAM is the major molecule serving as the capture antigen for CTC research reported in the literature [36-38]. EpCAM is an epithelial cell-specific marker which is highly expressed in breast cancer, prostate cancer and colon cancer but not in RCC (Figure 1) [39-41]. It is widely used as a CTC-capture antigen in many platforms, such as the CellSearch, microchip, microvortex-generating herringbone-chip, flow cytometry, magnetic sweeper and microfluidic devices [2, 42-49]. However, a growing body of research has indicated that EpCAM fails to recognize CTCs that are deficient in epithelial features, leading to low capture efficiency in such circumstances [7].

In this study, we proposed to substitute EpCAM with two cell surface markers, CA9 and CD147, for capturing RCC CTCs in peripheral blood. CA9 is a tumor hypoxia marker widely expressed in various types of human cancer [50-52]. High expression of CA9 in tumors is associated with increased aggressiveness and poor prognosis [53]. Specifically in RCC, CA9 is strongly expressed on the cell membrane [54] and associated with the histological subtype of specimens, with higher expression in CCRCC than in other RCC subtypes [17]. CD147 has also long been implicated in cancer development and metastasis with a close association with lymph node metastasis by enhancing EMT cell invasion through activation of the MAPK/ERK pathway [55]. CD147 expression is positively correlated with TNM stage and poor prognosis in RCC, with particularly high expression in advanced RCC that display higher T stage and shorter survival time [21, 56, 57]. Our findings on the prevalent expression of both CA9 and CD147 in our RCC patient cohort are consistent with previous reports from other groups (Figure 1 and Table 2). Although the idea of using CA9 and CD147 to attract CTCs is new, the characteristics of these molecules along with their high expression in RCC suggest their potential use as RCC CTC-capture antigens [53, 58, 59], which is supported by our findings that the combined use of CA9 and CD147 as capture antigens retrieved CTCs from both CTC-mimicking renal cancer cells as well as RCC patient blood with significantly improved capture efficiency compared to the EpCAM-based capture method (Figure 3).

The clinical value of CTCs has been extensively explored in a spectrum of cancers in recent years [2]. As reported by Bluemke *et al*, the patient CTC count was an independent prognostic factor correlating with lymph node invasion in RCC [60]. In another study, Seideman *et al* succeeded in capturing at least one CTC from 11 out of 12 RCC patients by the CellSearch system, but the CTC count failed to correlate with pathologic outcomes because of the insufficient number of patients studied [61]. In the present study, we were able to use the NanoVelcro platform in combination with the CA9-/CD147-based enrichment method to isolate CTCs from 72 out of 76 (94.7%) RCC patients. Our new CTC-capture system exhibited significantly higher RCC CTC-capture efficiency than other approaches previously reported elsewhere. Our results further demonstrated the potential prognostic value of captured CTCs by both CTC counts and CTC molecular marker expression (Figure 5 and Table 4).

In addition to CK, the gold standard marker defining epithelial CTCs, emerging studies have characterized CTCs with different molecular markers to monitor diverse cellular events that CTCs undergo in phenotypic changes [62]. A transition of adherent epithelial cells to a migratory mesenchymal state has been implicated in tumor metastasis and chemo-resistance in preclinical models of multiple cancers [63-66]. Recent studies have also characterized EMT in CTC platforms from breast cancer patients and revealed dynamic changes in the epithelial and mesenchymal composition in CTCs. One intriguing finding was that mesenchymal cells were highly enriched in CTCs, which was associated with disease progression [67]. In line with these reports, we demonstrated the expression of Vimentin, a mesenchymal marker, in clinical RCC samples by IHC (data not shown) as well as in CTCs captured from peripheral blood (Figure 4). Moreover, we showed that the CTC expression of Vimentin was capable of stratifying clinical stages in our RCC cohort, providing a more stringent correlation of CTC number with disease progression than standard CTC enumeration methods (Figure 5 and Table 4). Given the growing interest in CTC research going beyond CTC counts, further molecular characterization of CTCs merits more attention, which would benefit from the ongoing development of improved capture platforms, such as the NanoVelcro system, aiming eventually for the noninvasive real-time monitoring of molecular changes in a “liquid biopsy” to allow clinicians to execute individually tailored treatment strategies.

In summary, we successfully developed a set of RCC CTC-capture antigens combining the use of cell surface markers CA9 and CD147 to capture CTCs in RCC patients. We showed for the first time that this enrichment strategy in combination with the NanoVelcro chip system dramatically improved the CTC-capture efficiency in RCC compared to the conventional EpCAM-based approach. Importantly, we demonstrated the clinical association of CTC number and Vimentin expression status in CTCs with RCC disease progression, including pathologic feature and clinical stage, which suggests the prognostic value of CTCs in RCC. Together, these studies provide new insights into developing cancer type-specific capture antigens for CTC isolation with improved efficacy, making CTCs a valuable addition to the armamentarium for cancer detection, prognosis and treatment.

## MATERIALS AND METHODS

### Ethics statement

Written informed consents were obtained from all patients. All data used in this study have been anonymized. This study was approved by the Ethics Committee (IRB) of Xijing Hospital (Xi'an, Shaanxi, China) (see Table 1 for detailed patient information) and performed in accordance with relevant Chinese regulations and the Declaration of Helsinki [68].

### Patients

A total of 76 RCC patients, together with 10 benign renal patients, including angiomyolipoma, renal adenoma and fibroma, and 15 healthy volunteers were consecutively recruited from Xijing Hospital. All RCC patients (n=76) were staged and grouped according to the 2010 American Joint Committee on Cancer TNM classification for RCC [69]. The clinical and histopathologic characteristics of the patients enrolled in this cohort are described in Tables 1 and 2.

### NanoVelcro chip fabrication

The NanoVelcro chips were fabricated following the well-established method reported by Wang *et al* [70]. The NanoVelcro chip is composed of three parts: (i) a patterned silicon nanowire (SiNW) substrate coated with streptavidin, (ii) an overlaid polydimethylsiloxane (PDMS) chaotic mixer, and (iii) a home-machined holder set to sandwich a well-aligned PDMS mixer chip with the SiNW substrate.

### CTC capture from patient blood samples

For each participant, a total volume of 2 mL peripheral blood was collected using an EDTA-containing vacutainer tube. The blood was first treated with lymphocyte separation medium (Dingguo, Beijing, China) to remove red blood cells. Mono-nucleated cells were rinsed with PBS and then standard culture medium after centrifuging at 600 g for 10 minutes. The supernatant was discarded and the cell pellet was re-suspended in 200 μL 2% donkey serum. Before loading the sample, the streptavidin-coated (1 mg/mL, Life Technologies, Carlsbad, USA) NanoVelcro chip was modified with anti-CA9 (0.1 μg/mL, BAF2188, R&D Systems, Minneapolis, USA) and anti-CD147 (0.1 μg/mL, BAF972, R&D Systems) antibodies by loading antibody solution into the channel using a syringe pump (KDS 200, KD Scientific Inc., Boston, USA). Prior to sample injection, the channel underwent PBS wash for multiple times to remove free antibodies. Afterwards, the prepared cell suspension was loaded at a 0.5 mL/h flow rate. Finally, the NanoVelcro chip was loaded with 2% paraformaldehyde (PFA) for CTC fixation for further analysis.

### IHC analysis of tumor specimens

In this study, we obtained 70 surgical tissue samples from the 76 registered RCC patients. Paraneoplastic normal renal tissues were used as negative control. Formalin-fixed paraffin-embedded samples were prepared as 4 mm-thick sections and subjected to IHC staining using our previously published protocol [71]. After being blocked with 10% (v/v) goat serum for 30 minutes, slides were immersed in anti-EpCAM antibody (dilution 1:200; ZSGB, Beijing, China), anti-CA9 antibody (dilution 1:200; Santa Cruz Biotechnology, Santa Cruz, USA), or anti-CD147 antibody (dilution 1:200; ZSGB) diluted in PBS containing 1% (w/v) bovine serum albumin (BSA) at 4°C overnight in a moist chamber. After being treated with corresponding secondary antibodies for 30 minutes, the slides were stained with diaminobenzidine (DAB, ZSGB) and counterstained with hematoxylin. A light microscope (Olympus BX51, Olympus, Japan) was used to capture images with a DP70 digital camera.

IHC staining in each sample was scored by a semi-quantitative method by taking into account both staining intensity (I) and quantity for the proportion of tumor cells stained (q) to obtain a final score defined as the product of I × q. The scoring system for I was: 0, negative; 1, weak; 2, moderate; 3 strong staining. The scoring system for q was: 0, <1%; 1, 2-25%; 2, 26-50%; 3, 51-75%; 4, 76-100%. Finally, IHC scores ranging from 0 to 12 were obtained and further categorized into two groups, negative (-, 0-2) and positive (+, 3-12), based on different scores. All scoring was performed by two pathologists independently who had no prior information on the pathologic status of specimens to be assessed.

### Cell culture

Human renal cancer Caki-1 and Caki-2 cell lines were obtained from the American Type Culture Collection (ATCC, Manassas, USA). Cells were cultured in RPMI1640 (Life Technologies, Carlsbad, USA) supplemented with 5% fetal bovine serum (FBS, Life Technologies), penicillin (100 unit/mL), and streptomycin (100 μg/mL) at 37°C in a humidified atmosphere with 5% CO_2_. Before spiking, cells were detached with trypsin (Life Technologies), washed and suspended in PBS.

### Immunocytochemistry and immunofluorescence analysis of CTCs

A 200 μL primary antibody cocktail consisting of 1 μL rabbit anti-pan CK antibody (ab9377, Abcam, Cambridge, UK), 2 μL mouse anti-CD45 (ab30470, Abcam), 2 μL chicken anti-Vimentin antibody (ab24525, Abcam), 20 μL 10× donkey serum and 175 μL PBS were added into the CTC fixation substrate and incubated at 4°C overnight. To confirm the expression of CA9 and CD147 in captured CTCs, alternative primary antibody cocktail by replacing aforementioned anti-pan CK and anti-Vimentin antibodies with anti-CA9 and anti-CD147 antibodies as described in IHC analysis of human RCC samples was used. The CTC fixation substrates were then washed with PBS twice, and a 200 μL secondary antibody cocktail consisting of 4 μL Alexa Fluor 488 conjugated donkey anti-rabbit antibody (A21206, Life Technologies), 4 μL Alexa Fluor 555 conjugated donkey anti-mouse antibody (A31570, Life Technologies), 4 μL Alexa Fluor conjugated 647 goat anti-chicken IgG antibody (ab150175, Abcam), 20 μL 10× donkey serum and 168 μL PBS were added into the CTC fixation substrate and incubated at room temperature in the dark for 1 hour. After applying DAPI (1:1000, Life Technologies) for nuclear staining, the sections were mounted with a cover slip for fluorescence microscopy. Images were obtained by a light microscope equipped with NIS-Element imaging software (ECLIPSE 90i, Nikon, Japan). In brief, the whole area of the NanoVelcro chip was first scanned at 40× magnification, and the images were analyzed for potential cells identified in 400× magnification. The final enumerations were based on the criteria of DAPI^+^/CK^+^/CD45^−^, 13 μm< cellular diameter <50 μm, and nuclear/cytoplasmic ratio >50%.

### Statistical analysis

Statistical analysis was performed with SPSS 17.0 software (SPSS Inc., Armonk, USA). Statistical comparisons were analyzed using Student's t test, and *p* values less than 0.05 were considered to be statistically significant.
